# Transforming Growth Factor-β1 (TGF-β1) Induces Mouse Precartilaginous Stem Cell Proliferation through TGF-β Receptor II (TGFRII)-Akt-β-Catenin Signaling

**DOI:** 10.3390/ijms150712665

**Published:** 2014-07-17

**Authors:** Li Cheng, Chengyu Zhang, Ding Li, Jian Zou, Junfang Wang

**Affiliations:** Department of Orthopedics, Wuxi People’s Hospital Affiliated to Nanjing Medical University, Wuxi 214023, China; E-Mails: zhangruiwx11@163.com (L.C.), chengyuzhangas@126.com (C.Z.), dingdingfl163@163.com (D.L.)

**Keywords:** precartilaginous stem cells, chondrogenesis, TGF-β1, proliferation, Akt, β-catenin and signaling

## Abstract

Precartilaginous stem cells (PSCs) could self-renew or differentiate into chondrocytes to promote bone growth. In the current study, we aim to understand the role of transforming growth factor-β1 (TGF-β1) in precartilaginous stem cell (PSC) proliferation, and to study the underlying mechanisms. We successfully purified and primary-cultured PSCs from the neonate mice’ perichondrial mesenchyme, and their phenotype was confirmed by the PSC marker fibroblast growth factor receptor-3 (FGFR-3) overexpression. We found that TGF-β1 induced Akt-glycogen synthase kinase-3β (GSK3β) phosphorylation and β-catenin nuclear translocation in the mouse PSCs, which was almost blocked by TGF-β receptor-II (TGFRII) shRNA knockdown. Further, perifosine and MK-2206, two Akt-specific inhibitors, suppressed TGF-β1-induced GSK3β phosphorylation and β-catenin nuclear translocation. Akt inhibitors, as well as β-catenin shRNA knockdown largely inhibited TGF-β1-stimulated cyclin D1/c-myc gene transcription and mouse PSC proliferation. Based on these results, we suggest that TGF-β1 induces Akt activation to promote β-catenin nuclear accumulation, which then regulates cyclin D1/c-myc gene transcription to eventually promote mouse PSC proliferation.

## 1. Introduction

The application of chondrocytes in clinical settings is restrained due to the poor renewing ability [1,2]. Precartilaginous stem cells (PSCs) could self-renew or differentiate into chondrocytes to promote bone growth [2,3,4]. Robinson * et al.* have isolated PSCs from perichondrial mesenchyme (also termed “the ring of La Croix”) of neonate rats by immunomagnetic beads through fibroblast growth factor receptor-3 (FGFR-3) antibody selection [3]. These PSCs have potential to proliferate and to differentiate directionally into chondrocytes [3,4]. Transforming growth factor-β1 (TGF-β1) is shown to promote adult stem cell proliferation and chondrocyte differentiation [5,6], while its role in PSC proliferation and the underlying signaling mechanisms are not studied.

TGF-β binds to the type I and type II receptors on the cell surface, and TGF-β receptor II (TGFRII) phosphorylates the TGF-β receptor I (TGFRI) kinase domain, leading to Smad protein phosphorylation and activation [7]. The activated Smad complexes then translocate into the nuclei and regulate the transcription of target genes [7,8]. Meanwhile, TGF-β1 could also activate the non-canonical signaling pathways (also termed “non-Smad pathways”) [9]. For example, TGF-β1 is known to activate the Erk/MAPK [10,11] pathway and the phosphoinositide 3-kinase (PI3K)/Akt [12,13,14,15] pathway. These non-Smad pathways work independently or together with Smad complexes to regulate TGF-β1’s functions [7,8,10,11,12,13,14]. For example, activation of Akt signaling by TGF-β1 is shown to promote cell proliferation [16,17,18].

The transcription factor β-catenin is the key player in Wnt signaling [19,20,21,22]. Without Wnt ligand stimulation, cytosol β-catenin is phosphorylated and degraded through ubiquitination [23]. Upon Wnt stimulation, Wnt molecules binding to its membrane-bound receptor (Frizzled) and the co-receptor (LRP5/6) [20,24,25,26], then the kinases (*i.e*., glycogen synthase kinase-3β (GSK3β) and adenomatous polyposis coli (APC)) [27] that phosphorylate and destabilize β-catenin are inhibited; thus, β-catenin accumulates in the nucleus, where it associates with transcription factors, such as T-cell factor (TCF)/lymphoid enhancing factor(LEF) [28] to activate transcription of Wnt-responsive genes (*i.e.*, cyclin D1 and c-myc) [29,30,31], which are important for self-renewing (proliferation) and differentiation [29,30,31]. Recent studies have identified a cross-talk between the TGF-β and β-catenin signaling pathways in adult and embryonic stem cells [32,33]. It was found that TGF-β1 could directly promote β-catenin nuclear translocation without affecting β-catenin stability or phosphorylation [32,33]. In the current study, we first isolated, purified and cultured PSCs from the perichondrial mesenchyme of neonate mice and then explored the potential role of TGF-β1 in mouse PSC proliferation by focusing on the signaling mechanisms. We discovered that TGF-β1 induces mouse PSC proliferation through TGF-β receptor II (TGFRII)-Akt-β-catenin signaling.

## 2. Results

### 2.1. Mouse Precartilaginous Stem Cell (PSC) Isolation and Culture

**Figure 1:**
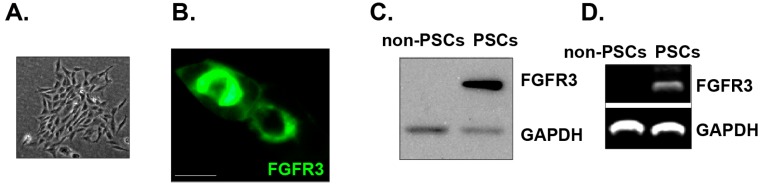
Mouse precartilaginous stem cell (PSC) isolation and culture. The morphology of mouse precartilaginous stem cells (PSCs) at Day 4 of culture are shown (**A**); Immunofluorescence microscopy (**B**); western blotting (**C**) and RT-PCR (**D**) results showed the expression of FGFR-3 in cultured mouse PSCs (Day 4 of culture), while the cells left after immunomagnetic separation (termed “non-PSCs”) were negative for FGFR-3. GAPDH was tested as the loading control (**C**, **D**). Magnification: 1:200 (**A**). Bar = 15 μm (**B**). Experiments in this figure were repeated three times, and similar results were obtained.

Using the method described, we successfully isolated and purified the precartilaginous stem cells (PSCs) from the perichondrial mesenchyme (the La Croix rings) of the neonate mice. The morphology of mouse PSCs at Day 4 of culture is shown in Figure 1A. As discussed early, studies have been using FGFR-3 as a marker for PSCs [2,3]. We thus tested FGFR-3 expression in the immunomagnetic bead-selected mouse PSCs. The immunofluorescence image in Figure 1B confirmed FGFR-3 expression in the mouse PSC plasma membrane. Further, RT-PCR and western blotting results confirmed FGFR-3 mRNA and protein expression in the mouse PSCs (Figure 1C,D). Note that RT-PCR and western blotting results showed no FGFR-3 expression in the cells left after immunomagnetic separation (non-PSCs) (Figure 1C,D).

### 2.2. TGF-β Receptor-II Is Required for TGF-β1-Induced Akt/GSK3β Phosphorylation and β-Catenin Nuclear Translocation

**Figure 2:**
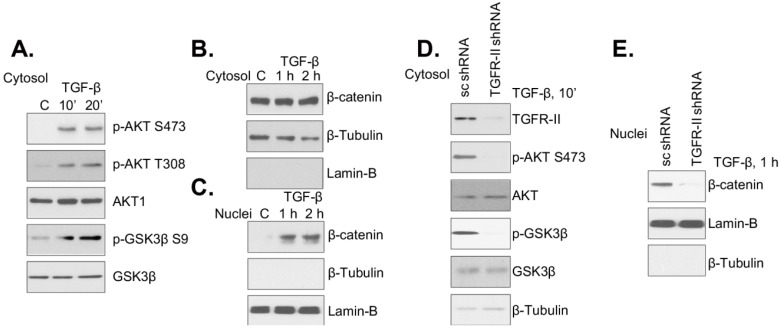
TGF-β receptor-II is required for TGF-β1-induced Akt/GSK3β phosphorylation and β-catenin nuclear translocation. Mouse PSCs (Day 4) were treated with TGF-β1 (TGF-β, the same for all figures, 25 ng/mL) for the indicated time point; cytosol and nuclear fractions were isolated, and the expression of indicated proteins in the corresponding fraction was tested by western blotting (**A**–**C**). The lentiviral particles containing TGFRII-shRNA or scramble-shRNA (15 μL/mL each) were added to mouse PSCs (Day 4) for 48 h. Afterwards, mouse PSCs were treated with TGF-β1 (25 ng/mL) for the indicated time point; cytosol and nuclear fractions were isolated, and the expression of indicated proteins in the corresponding fraction was tested by western blotting (**D**, **E**). Experiments in this figure were repeated three times, and similar results were obtained. “C” stands for the PBS control.

Here, we tested the potential role of TGF-β1 on PSC proliferation. We first examined the response of mouse PSCs to TGF-β1. Western blotting results in Figure 2A show that TGF-β1 (25 ng/mL) induced significant Akt/GSK3β phosphorylation in cultured mouse PSCs. Meanwhile, the same concentration of TGF-β1 promoted β-catenin nuclear translocation (Figure 2C), without affecting its overall expression (Figure 2B). We then explored the involvement of TGF-β receptor-II (TGFRII) in TGF-β1 signaling. The TGFRII-shRNA containing lentiviral particles were applied to selectively knockdown TGFRII in cultured mouse PSCs. Western blotting results demonstrated that TGFRII was dramatically downregulated in PSCs after TGFRII-shRNA lentiviral infection, while cells infected with scramble-shRNA lentivirus showed intact TGFRII expression (Figure 2D). Significantly, TGFRII knockdown dramatically inhibited TGF-β1-induced Akt/GSK3β phosphorylation (Figure 2D) and β-catenin nuclear translocation (Figure 2E), indicating the requirement of TGFRII in TGF-β1 signaling in mouse PSCs.

### 2.3. Akt Blockers Inhibit TGF-β1-Induced GSK3β Phosphorylation, β-Catenin Nuclear Translocation and Cell Proliferation in Mouse PSCs

**Figure 3:**
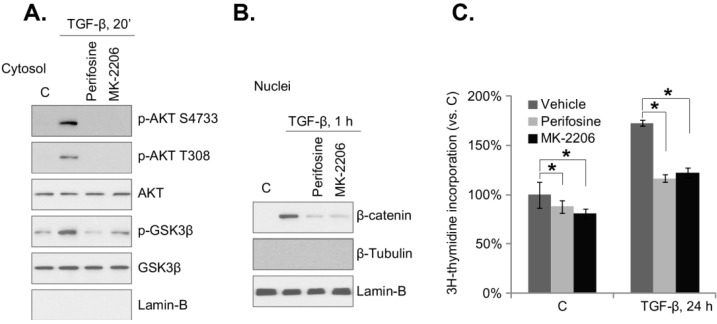
Akt blockers inhibit TGF-β1-induced GSK3β phosphorylation, β-catenin nuclear translocation and cell proliferation in mouse PSCs. Mouse PSCs were pre-treated with perifosine (2.5 μM) or MK-2206 (5 μM) for one hour, followed by TGF-β1 (25 ng/mL) stimulation. Cells were further cultured for the indicated time point; cytosol and nuclear fractions were isolated, and the expression of the indicated proteins in the corresponding fraction was tested by western blotting (**A**, **B**); Cell proliferation was tested by the ^3^H-thymidine incorporation assay (**C**). Experiments in this figure were repeated three times, and similar results were obtained. * *p* < 0.05 “C” stands for the PBS control.

The β-catenin level is kept low by a continued process of phosphorylation-dependent ubiquitination and degradation [20,22,34,35]. When the kinases that phosphorylate and destabilize β-catenin are inhibited, β-catenin will travel to and accumulate in the nuclei, where it associates with TCF/LEF transcription factors to activate its responsible genes [20,22,34,35]. Akt is known to phosphorylate and inhibit GSK-3β, which could allow β-catenin to translocate to the nuclei. We have shown that TGF-β1-induces GSK-3β in-activation (phosphorylation) in mouse PSCs (Figure 2); we then tested the involvement of Akt in this process. As expected, two Akt-specific inhibitors, perifosine [36] and MK-2206 [37], blocked TGF-β1-induced Akt activation (Ser 473 and Thr 308 phosphorylation) in mouse PSCs (Figure 3A). Significantly, TGF-β1-induced GSK3β phosphorylation and β-catenin nuclear translocation were also suppressed by the Akt inhibitors (Figure 3A,B), indicating that Akt activation is important for TGF-β1-induced β-catenin nuclear translocation. Studies have found that β-catenin nuclear translocation could promote cell proliferation [21,24,34,38,39]. Here, the ^3^H-thymidine incorporation assay results showed that TGF-β1 promoted mouse PSC proliferation, which was inhibited by perifosine and MK-2206. Note that basal mouse PSC proliferation was also inhibited by the above Akt inhibitors.

### 2.4. β-Catenin Silencing Inhibits TGF-β1-Induced Mouse PSC Proliferation

**Figure 4:**
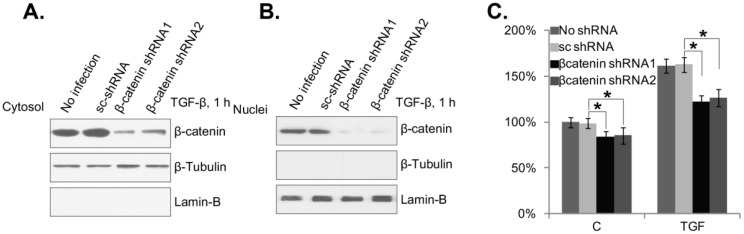
β-catenin silencing inhibits TGF-β1-induced mouse PSC proliferation. The lentiviral particles containing different β-catenin-shRNAs (targeting non-overlapping sequence, -1/-2) or scramble-shRNA (15 μL/mL each) were added to mouse PSCs (Day 4) for 48 h. Afterwards, mouse PSCs were treated with TGF-β1 (25 ng/mL) for one hour; cytosol and nuclear fractions were isolated, and the expression of indicated proteins in the corresponding fraction was tested by western blotting (**A**, **B**); The above PSCs were also treated with TGF-β1 (25 ng/mL) for 24 h, and cell proliferation was tested by the ^3^H-thymidine incorporation assay (**C**). Experiments in this figure were repeated three times, and similar results were obtained. * *p* < 0.05 “C” stands for the PBS control.

To explore the role of β-catenin in mouse PSC proliferation by TGF-β1, we utilized β-catenin-shRNA containing lentiviral particles to knockdown β-catenin. Two non-overlapping β-catenin-shRNAs were applied here. Western blotting results in Figure 4A showed that both shRNAs efficiently downregulated β-catenin expression in mouse PSCs. Correspondingly, TGF-β1-induced β-catenin nuclear translocation was also inhibited by the shRNAs (Figure 4B). Meanwhile, mouse TGF-β1-induced PSC proliferation was also inhibited when β-catenin was silenced (Figure 4C). PSC basal proliferation was also inhibited by β-catenin silencing, further suggesting the role of β-catenin in PSC proliferation (Figure 4C). Thus, these results indicate that β-catenin nuclear translocation is important for TGF-β1-induced mouse PSC proliferation.

### 2.5. Akt Activation and β-Catenin Are Important for TGF-β1-Induced Cyclin D1/C-Myc Transcription in Mouse PSCs

**Figure 5:**
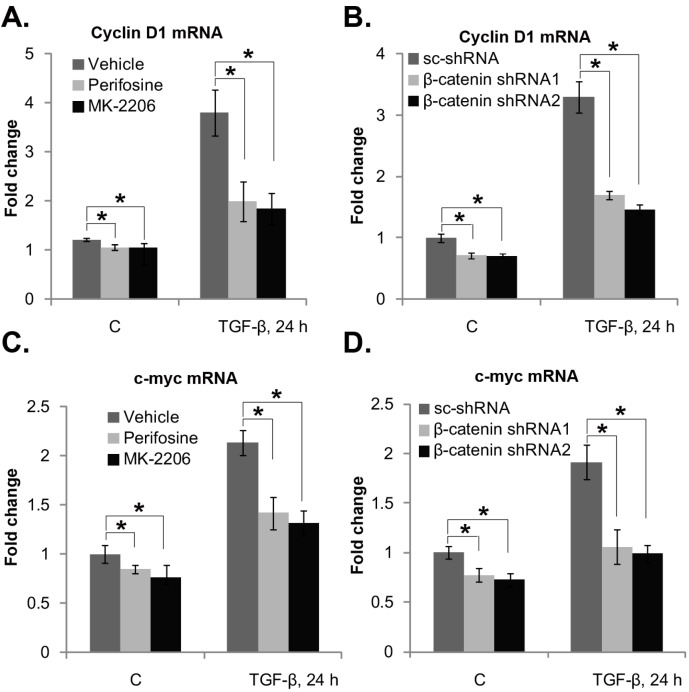
Akt activation and β-catenin are important for TGF-β1-induced cyclin D1/c-myc transcription in mouse PSCs. Mouse PSCs were pre-treated with perifosine (2.5 μM) or MK-2206 (5 μM) for one hour, followed by TGF-β1 (25 ng/mL) stimulation. Cells were further cultured, after 24 h, and the mRNA expression of cyclin D1 (**A**) and c-myc (**C**) was tested by real-time PCR. The lentiviral particles containing β-catenin-shRNA-1, β-catenin-shRNA-2 or scramble-shRNA (15 μL/mL each) were added to mouse PSCs (Day 4) for 48 h; afterwards, cells were treated with TGF-β1 (25 ng/mL). Cells were further cultured for 24 h, and the mRNA expression of cyclin D1 (**B**) and c-myc (**D**) was tested by real-time PCR. Experiments in this figure were repeated three times, and similar results were obtained. * *p* < 0.05. “C” stands for the PBS control. Vehicle stands for 0.01% DMSO.

We have shown that TGF-β1-TGFRII activates Akt to inhibit GSK3β, whiling inducing β-catenin nuclear translocation. Meanwhile, Akt-dependent β-catenin nuclear translocation is important for TGF-β1-induced PSC proliferation. Among β-catenin regulated genes, cyclin D1 [29,30] and c-myc [31] are critical for cell proliferation. Thus, we tested the effect of TGF-β1 on cyclin D1 and c-myc transcription in cultured mouse PSCs. Real-time PCR results in Figure 5 showed that TGF-β1 induced significant cyclin D1 and c-myc mRNA expression in cultured mouse PSCs. Significantly, Akt inhibitors (perifosine or MK-2206) (Figure 5A,C), as well as β-catenin shRNAs silencing (Figure 5B,D) significantly inhibited TGF-β1’s effect on those two genes in mouse PSCs. Thus, these results suggest that Akt activation and β-catenin are important for TGF-β1-induced cyclin D1/c-myc mRNA expression in mouse PSCs.

## 3. Discussion

Through immunomagnetic beads, we are the first to successfully isolate, purify and culture PSCs from the perichondrial mesenchyme of neonate mice. We also tested the biological functions of TGF-β1 in the mouse PSCs and demonstrated, for the first time, that TGF-β1 activated TGFRII to induce cyclin D1/c-myc gene transcription to promote mouse PSC proliferation. For the mechanism study, we discovered that TGF-β1 induced β-catenin nuclear accumulation, which was required for mouse PSC proliferation. Significantly, we confirmed that TGF-β1-induced β-catenin nuclear accumulation, and mouse PSC proliferation was surprisingly due to the activation of a non-canonical TGF-β signaling pathway: Akt-GSK-3β signaling.

Here, we propose the existence of signaling cross-talk between the TGF-β and β-catenin signaling pathways in cultured mouse PSCs. Our results showed that TGF-β1 induces GSK-3β phosphorylation through the AKT pathway, which induces β-catenin stabilization and nuclear translocation. TGF-β1 failed to change the expression of β-catenin in PSCs. Rather; we found that the effector of TGF-β1 signaling, in particular the activation of Akt signaling, plays an essential role in shuttling β-catenin into the nucleus. One possible mechanism is that TGF-β1 activates the Akt pathway to inhibit GSK-3β through phosphorylation, which allows β-catenin to translocate to the nucleus, activating TCF/LEF-dependent transcription. To support this hypothesis, we observed GSK-3β phosphorylation/in-activation by TGF-β1, while Akt inhibitors blocked TGF-β1-induced GSK-3β phosphorylation and β-catenin nuclear translocation.

The activation of β-catenin is reported to be associated with enhanced proliferation, and well-known target genes of β-catenin, such as c-myc [31] and cyclin D1 [29,30], encode important positive regulators of cell proliferation. In the current study, we found that TGF-β1 induced the transcription of c-myc and cyclin D1 genes and promoted mouse PSC proliferation. These effects by TGF-β1 were largely alleviated by β-catenin silencing. Further, Akt inhibitors (including perifosine [36] and MK-2206 [37,40,41]), which blocked TGF-β1-induced β-catenin nuclear accumulation, also suppressed TGF-β1-induced c-myc and cyclin D1 transcription, as well as PSC proliferation. Thus, we suggest that TGF-β1-induced PSC proliferation might require the Akt-dependent and β-catenin-regulated transcription of proliferation genes (*i.e.*, c-myc and cyclin D1).

## 4. Materials and Methods

### 4.1. Chemicals, Reagents and Antibodies

TGF-β1, perifosine and MK-2206 were obtained from Selleck (Shanghai, China). Anti-Akt1, GSK3β, S6K1, FGFR3, β-catenin and glyceraldehyde-3-phosphate dehydrogenase (GAPDH) antibodies were obtained from Santa Cruz Biotechnology (Santa Cruz, CA, USA). All other kinase antibodies used in this study were obtained from Cell Signaling Technology (Shanghai, China). 

### 4.2. Precartilaginous Stem Cells Isolation, Purification and Culture

Similar to previous studies [3,4], the neonate C57BL/6J mice were provided by the animal center of Nanjing Medical University. The tissues located around the perichondrial mesenchyme (also termed “the La Croix rings”) were cut down and digested sequentially with Complete Trypsin Solution (Chemicon International Inc., Temecula, CA, USA) with 0.05% collagenase type I (Sigma Chemical Co., St. Louis, MO, USA). After the cells were dispersed and suspended as a single cell suspension in 0.1 M phosphate buffer saline (PBS), they were incubated with FGFR-3 antibody (E-7) (1:500, Santa Cruz Biotechnology Inc., Santa Cruz, CA, USA) [3] and then purified by an immunomagnetic separation system (Miltenyi Biotech, Bergisch Gladbach, DE, Germany). The immuno-selected mouse precartilaginous stem cells (PSCs) were then cultured in DMEM/F12 medium (Thermo Fisher Scientific Inc., Fremont, CA, USA), supplemented with 20% fetal calf serum (FCS, Gibco, Shanghai, China), 100 units/mL penicillin and streptomycin in a 5% CO_2_/37 °C incubator. The detailed procedures have been described [3,4]. The medium was switched every 2 days. The protein and mRNA expression of FGFR-3 were tested by Western blotting and RT-PCR to verify the phenotype of the PSCs.

### 4.3. FGFR-3 Immunofluorescence in Mouse PSCs

The purified mouse PSCs were seeded into six-well-plates with 5 × 10^5^ cells/well. After attachment, mouse PSCs were fixed with 4% paraformaldehyde for 20 min at room temperature. The cells were then permeabilized with 0.2% Triton X-100 solution for 10 min at 4 °C. Cells were then incubated with the rabbit-anti-FGFR-3 (1:200 dilution, Santa Cruz Biotech, Santa Cruz, CA, USA) at 4 °C overnight. Next, the detection of the bound primary antibodies was enabled by incubating cells with goat anti-rabbit IgG-Cy3 (Cellular Signaling Tech, Shanghai, China) for 1 h at 37 °C; the cells were then observed, and images were recorded under an Olympus fluorescence microscope (CX41, Olympus, Tokyo, Japan).

### 4.4. Western Blotting

Mouse PSCs were washed twice with ice-cold PBS and then lysed using lysis buffer, which contained 1% Nonidet P-40 (NP-40), 1% deoxycholate, 0.1% sodium dodecyl sulfate, 150 mmol/L sodium chloride and 10 mmol/L Tris-HCl (pH, 7.4). The lysates were then collected and centrifuged. The concentration of the extracted protein was measured by a bicinchoninic acid assay kit (Sigma, Shanghai, China). The extracted protein was boiled for 5 min in loading buffer. Samples (20–30 μg/well) were separated by the 10% SDS-polyacrylamide gel, and after electro-blotting onto polyvinylidene fluoride (PVDF) membranes (Millipore, Shanghai, China), the blots were blocked with blocking solution (10% (*w*/*v*) milk in Tris-buffered solution plus Tween-20 (TBST)), incubated overnight at 4 °C with primary antibodies and then incubated with HRP-conjugated anti-rabbit/mouse secondary antibodies. The detection was performed by Super-signal West Pico Enhanced Chemiluminescent (ECL) Substrate. The nuclei of PSCs were isolated by the nuclei isolation kit purchased from Sigma (Shanghai, China), based on the instructions provided.

### 4.5. ^3^H-Thymidine Incorporation Assay

The mouse PSCs were seeded at a density of 5 × 10^4^ cells/well in 0.5 mL DMEM containing 10% FCS onto the 48-well tissue culture plates; cells were serum-starved overnight before being exposing to TGF-β1 with other treatment/s for 24 h, in the presence of 1 μCi/ml of tritiated thymidine. To determine ^3^H-thymidine incorporation, PSCs were washed with PBS (4 °C) 3–4 times; the DNA was then precipitated with cold 10% trichloroacetic acid, solubilized with 1.0 M sodium hydroxide, and aliquots were counted by liquid-scintillation spectrometry. The value was always normalized to the control group.

### 4.6. Total RNA Isolation and Real-Time Reverse Transcriptase Polymerase Chain Reaction (RT-PCR)

Total RNA was prepared by RNA-TRIZOL extraction (Gibco, Nanjing, China). The concentration and the purity of the extracted RNA were measured spectrophotometrically at A260 and A280. Real-time reverse transcription-polymerase chain reaction (real-time PCR) was performed byusing the TOYOBO ReverTra Ace RT-PCR kit according to the manufacturer’s instructions. The primers were F: 5'-AAGCTGTGCATCTACACCGA-3'/R: 5'-CTTGAGCTTGTTCACCAGGA-3'; for mouse *cyclin D1* [42]; F: 5'-AGGATAAAGTCTAGGTCCAGGAGGTCGTTG-3' R: 5'-AGTCGTAGTCGAGGTCATAGTTCCTGTTGG-3' for mouse *c-myc* [43]; 5'-GAAGGTGAAGGTCGGAGTC-3'/R: 5'-GAAGATGGTGATGGGATTTC-3' for mouse *GAPDH* and F: 5'-CGCTTTGCTGAGGTCTATAAGGC-3'/R: 5'-GATATTGGAGCTCTTGAGGTCCCT-3' for mouse *TGFRII*. A typical reaction (50 μL) contained 1/50 of reverse transcription-generated cDNA and 200 nM of primer in 1× SYBR Green Real-Time Master Mix (Toyobo, Shanghai, China) buffer. The PCR reactions were carried out on a Bio-Rad IQ5 multicolor detection system by using 2 μg of synthesized cDNA under the following conditions: 95 °C for 5 min, 40 cycles at 95 °C for 15 s, 60 °C for 15 s and 72 °C for 30 s. All real-time PCRs were performed at least in triplicate. The value was always normalized to the control group.

### 4.7. Target Protein shRNA-Knockdown through Lentiviral Infection

Mouse PSCs were seeded in a six-well plate in the growth medium. The lentiviral particles containing shRNA of the targeted gene or scramble-shRNA (Santa Cruz Biotech, Santa Cruz, CA, USA) were added to the cells (15 μL/mL medium). After 12 h, the lentiviral particles containing medium were replaced by fresh growth medium, and cells were further cultured for another 48 h. The expression of target protein and the equal loading in the infected cells was detected by western blotting. The TGFRII-shRNA lentiviral particles were purchased from Santa Cruz Biotech (Santa Cruz, CA, USA); two mouse β-catenin-shRNAs (targeting non-overlapping β-catenin sequence) lentiviral particles were designed and synthesized by Kaiji Biotech (Shanghai, China).

### 4.8. Data Analysis

Data were collected using a minimum of three experiments and used to calculate the mean ± S.D. Statistical differences were analyzed by one-way ANOVA, followed by multiple comparisons performed with the *post hoc* Bonferroni test (SPSS version 18). Values of *p* < 0.05 were considered statistically significant.

## 5. Conclusions

In summary, the results of this study suggest that TGF-β1 activates Akt signaling to promote β-catenin nuclear accumulation, which then regulates cyclin D1/c-myc genes transcription to eventually promote mouse PSC proliferation.
